# The Ecology of Environmental Health

**DOI:** 10.1177/117863020800200001

**Published:** 2008-07-21

**Authors:** Timothy Kelley

**Affiliations:** Environmental Health Sciences Program, Department of Health Education and Promotion, College of Health and Human Performance, East Carolina University, Greenville, NC

The applied discipline of environmental health sciences seeks to clearly establish, secure and promote its role within many diverse science-based disciplines with similar names and yet very different approaches. These diverse disciplines include environmental science, ecology, environmental ethics, and many others. Traditionally, environmental health has been distinguished from these other disciplines in its emphasis on protecting human health (e.g. public and occupational health). Environmental protection is also an important part of environmental health, but has typically focused more on the affect that our environment has on human health rather than the effect that humans have on our environment. Environmental health also emphasizes practical, applied solutions to problems. One approach to this process integrates hazard *anticipation, recognition* and *evaluation* followed by intervention *identification, implementation* and *evaluation*. The intervention evaluation cycle is repeated until a problem is ultimately solved, or perhaps more realistically, until a higher priority problem takes precedent.

As an environmental health microbiologist completing my doctoral research in waste management in an ecological institute, and previously serving as faculty and adjunct faculty in biology departments, my personal environmental health experience is somewhat unusual. While I don’t identify myself as an ecologist, I believe that environmental health is beginning to recognize the value of an ecological systems approach to problem solving. Humans are just beginning to recognize, acknowledge and attempt to limit our contributions to a variety of environmental problems, with varying success. More people than ever before now seem to understand that the changes that we make in our environment yield results that affect our own health and well-being. To borrow a concept from the emminent biologist David Suzuki, we are all inextricably linked with our environment. Whatever we do within our environment ultimately affects us, either directly or indirectly, for better or worse. These affects have consequences on every scale, from our home environment and personal work-station ergonomics to global climate change and all of the intervening local, regional and national evironmental levels. Many humans no longer see ourselves in extreme terms, as either simply passive observers of our environment or as a plague upon it. We are recognizing and accepting that whenever we take a breath, drink, eat, touch, create waste or otherwise interact with our environment, we literally *become* a part of our environment. In fact, we have *always* been a part of our environment, but closer examination of these interactions serves to more clearly demonstrate this reality. The reductionist viewpoint of early science that the universe could be broken down into its essential elements and understood as simply the sum of its individual parts is now being recognized as hubris. The challenge is to see ourselves for what we truly are; one component in a vast, complex system from which we both extract resources and to which we contribute, both positively and negatively. Rather than focusing on a specific problem within this complex system, perhaps we can take a metaphorical step backward and examine the larger system that we inhabit with a wider view. In order to find the most effective and efficient environmental health intervention strategies, we should know as much as possible about the system we are affecting, within the ever-present practical restraints.

The Baltimore, Maryland journalist H.L. Mencken is credited with the quote: “There is always an easy solution to every human problem—neat, plausible, and *wrong*.” I interpret this to mean that complex problems often have complex solutions. Furthermore, even *simple* problems often don’t have simple solutions. When we seek to isolate one component of a system and “fix” it, we often create more problems (sometimes worse that the one we “solved”). The study of ecological systems introduces tremendous complexity, and brings to mind a quote attributed to the British chemist and statistician Charles Box: “All models are wrong, but some models are useful.” Models of how we interact with our environment allow us to view amazingly complex systems in simpler terms and can thereby help us to focus on the most important elements of the system. In fact, some have suggested that confusion arises because we have mistaken the model for reality. It takes care to avoid confusing the model with the system itself. Author Malcom Gladwell, in his bestselling books “The Tipping Point” and “Blink: The Power of Thinking Without Thinking” proposes that subtle, even unnoticed events have much more effect on outcomes than we imagine. A small shift (or intervention) in a system applied at the right time and the right “tipping point” can sometimes have significant postive or negative consequences. The best decisions are sometimes made intuitively in the blink of an eye, apparently with little rational thought. Unfortunately, this also applies to poor decisions. How can we examine the complex systems in which humans interact with our environment in order to identify and implement appropriate controls to reduce hazards and risks to human health and our environment? In order to be both effective and efficient in the long term, environmental health interventions should be applied at the highest scale that is possible and economically feasible. However, time, personnel, political and other restrictions often require that we compromise the ideal, in order to do the greatest practical good for the greatest number. This is where prioritzation becomes a key environmental health tool in order to apply interventions judiciously. A temporary intervention may require the use of personal protective equipment to protect workers from a hazard exposure in the short-term, but a better intervention would be to limit or eliminate the hazard from the workplace, and an even better one would be to replace it with a safe or less dangerous alternative on a regional or global scale.

A quality solution often addresses several problems at once. This may seem at first examination even more difficult than solving one problem at a time, but in fact, it takes into consideration the complex interactive nature of systems, even those that may appear simple when first examined. Rather than being simple, these solutions are sometimes referred to as “elegant”, which I interpret to mean that while they may appear to be simple, they in fact take into account elements which might have been overlooked or ignored by a more simplistic solution. On a more practical level, this systems approach to problem solving may require more in-depth consideration of the potential consequences of an intervention, through identifying both the positive and negative outcomes that may occur. A specific example might be the application of pollution prevention (P^2^) techniques to address waste management problems. While not addressing the “waste” (noun) directly, P^2^ addresses “waste” (verb) and thereby the root problem by eliminating or limiting waste generation before it ever becomes a problem. This type of problem solving is sometimes described as “thinking outside the box” (The origin of this phrase is obscure, but is thought to have come from Disney™ management, using a nine-dot puzzle “box” problem-solving exercise. The puzzle solution seems easy once demonstrated, but requires the solver to apply a new way of thinking about the box.) Thinking outside the box is simply recognizing that what we consider to be restrictions are often only those that we have imposed upon ourselves. Continuous evaluation, of course, is a key part of this process, providing not only less biased, more objective evidence for advantages and disadvantages to interventions, but suggesting modification of the process to maximize both its efficacy and efficiency.

The ecological concept of ecosystem diversity can be applied to environmental health “programmatic” areas such as air, water and food quality; waste management; toxicology and epidemiology, among others. As in ecology, these components serve to feed into and support one another to make environmental health stronger and more vigorous. This mutualistic, synergistic relationship provides a “robustness” to the system that strengthens all of its parts. Perhaps, as implied by biologist Lynn Margulis and others, humans may eventually evolve to develop a mutualistic relationship with our environment where humans and our environment both benefit from our interactions. Properly educated, well-trained environmental health professionals should consider not only the human health impacts of their decisions, but also the ecological, social, economic, political and psychological implications. Through our individual practice and research efforts, we contribute important pieces to this large, complex puzzle that may ultimately help us to gain new insights into the discipline of environmental health. This special edition of *Environmental Health Insights* seeks to share current developments in environmental health in a variety of programmatic areas. I invite you to consider while reading this special issue how these elements of environmental health may be integrated and to consider them from a more holistic, ecological perspective.

